# P-402. A Seven-Year Analysis of CRAB and CRE Urinary Tract Infections in Tennessee

**DOI:** 10.1093/ofid/ofae631.603

**Published:** 2025-01-29

**Authors:** Dipen Patel, Glodi Mutamba, Jacquelyn Taylor, Melphine Harriott, Daniel Muleta

**Affiliations:** HAI/AR, Tennessee Department of Health, Nashville, Tennessee; Tennessee Department of Health, Nashville, Tennessee; Vanderbilt University Medical Center, Nashville, Tennessee; TN Department of Health, Nashville, Tennessee; Tennessee Department of Health, Nashville TN, Antioch, Tennessee

## Abstract

**Background:**

Urinary tract infections (UTIs) are a prevalent health concern, affecting millions worldwide. The data collected through the Tennessee Multi-site Gram-negative Surveillance Initiative (MuGSI) was analyzed for a comparative study of UTIs caused by Carbapenem-Resistant *A. baumannii* complex (CRAB) and Carbapenem-Resistant *Enterobacterales* (CRE). We aimed to evaluate the demographics, prevalence, and clinical characteristics of patients with UTIs caused by CRAB and CRE.

**Methods:**

Tennessee has participated in the Center for Disease Control and Prevention (CDC) Emerging Infections Program MuGSI project since 2014. An incident case is defined as the first CRE of *E. coli, E. cloacae* complex*, Klebsiella* species or CRAB per patient within 30 days. We analyzed CRE and CRAB incident cases of primary or recurrent UTIs reported to MuGSI during 2016–2022. Descriptive analysis and odds ratio were performed using SAS 9.4.

**Results:**

From 2016–2022, 521 UTI cases were reported: 448 (86.0%) CRE and 73 (14.0%) CRAB. More males (64.4%) had CRAB infections (p< 0.0001); conversely, more females (68.5%) had CRE infections (p< 0.0001). The mean ages of patients with CRAB and CRE were 64 and 67 years, respectively. Incidence of CRAB was 8.6 in non-white and 3.3 in white per 100,000 population (p< 0.0001). CRE was associated with reduced odds (OR=0.17, p< 0.0001) of having indwelling catheters compared to CRAB. CRAB cases were 2.59 times more likely to be admitted to the intensive care unit (ICU) (p=0.0020). CRAB cases had higher odds of neurological conditions (OR=3.44, p< 0.0001), renal conditions (OR=2.27, p=0.0014), and diabetes (OR=1.67, p=0.0412). CRAB mortality was 6.9% compared to CRE at 2.0% (p=0.0178). Most CRAB and CRE cases were healthcare-associated infections (98.6% & 67.2%).

**Conclusion:**

The study marks the first multi-year evaluation of TN patients with CRE and CRAB UTIs. As expected, CRAB patients frequently needed ICU admission and were more likely to have indwelling catheters, neurological and renal conditions, and diabetes. This could be linked to ICU patient severity, but further studies are required to clarify. Additionally, significant race and sex gaps were noticed between CRAB and CRE-infected patients, prompting further investigation into these disparities.

**Disclosures:**

**All Authors**: No reported disclosures

